# Dyslipidaemia is common among patients with type 2 diabetes: a cross-sectional study at Tema Port Clinic

**DOI:** 10.1186/s13104-019-4245-9

**Published:** 2019-04-03

**Authors:** Emmanuel Kwaku Ofori, Dorcas Owusu-Ababio, Emmanuel A. Tagoe, Henry Asare-Anane

**Affiliations:** 1 Department of Chemical Pathology, School of Biomedical and Allied Health Sciences (S.B.A.H.S.), University of Ghana, Accra, Ghana; 2 Department of Medical Laboratory, Narh-Bita College, Tema, Ghana; 3 Department of Medical Laboratory, S.B.A.H.S, University of Ghana, Accra, Ghana

**Keywords:** Dyslipidemia, Diabetes mellitus, Cardiovascular, Transaminase

## Abstract

**Objective:**

This study aimed to evaluate dyslipidemia in Ghanaian subjects with type 2 diabetes.

**Results:**

Hundred individuals with type 2 diabetes and 61 apparently healthy controls participated. The prevalence of hypercholesterolemia among persons with type 2 diabetes was 53%. Blood pressure, fasting blood glucose (FBG), triglyceride (TG), low-density lipoproteins (LDL) and alanine transaminase (ALT) levels were higher in persons with type 2 diabetes compared with the control group (p < 0.01). Positive correlations were found within persons with type 2 diabetes for triglyceride vs FBG; ALT vs age and aspartate transaminase (AST) vs TG (p < 0.05 respectively). This study demonstrated hyperlipidemia and poor liver health in persons with type 2 diabetes.

## Introduction

Diabetes mellitus has become the fifth leading cause of death globally with increasing prevalence in the developing world [1, 2]. A prevalence rate of 3.3% has been estimated among Africans [3]. Uncontrolled diabetes mellitus leads to medical complications that include coronary risks, stroke, and damages to the extremities of the eyes, kidney and the nerves [4, 5].

A lipid profile is a direct measure of three blood components namely, total cholesterol (T. Chol), triglyceride (TG) and high-density lipoprotein (HDL) [6]. Other constituents such as low-density lipoproteins (LDL), atherogenic risk (AR), very low-density lipoproteins (VLDL) and coronary risk (CR) are usually derived from such direct measures [6, 7]. Lipid abnormalities have been implicated in the causation of insulin resistance, type 2 diabetes, obesity and atherosclerosis [8–12]. Studies in Ghana (albeit few), have estimated that between 3.3 and 6% of the general population has diabetes with the prevalence increasing with age and is higher in urban areas [13, 14]. The exodus from rural to urban communities continue to increase in Ghana [15, 16]. An earlier study at the Korle-Bu Teaching Hospital revealed that cardiovascular diseases (CVDs) constituted more than one-fifth of all causes of death from 2006 to 2010 [17]. Controlling levels of lipids could be a possible way of controlling high blood pressure and prevention of hypertensive nephropathy [18, 19]. Dyslipidemia may thus be more prevalent and may be playing a major role in the pathogenesis and management of diabetes mellitus. Little is known about the lipid profile pattern of Ghanaian persons with type 2 diabetes. Due to lifestyle risks associated with urbanization [13], this study aimed to evaluate dyslipidemia in Ghanaian patients with type 2 diabetes mellitus reporting at the Tema Port Clinic. It is anticipated that this study will provide information that could help in the management dyslipidemia among Ghanaian subjects.

## Main text

### Methods

The study design was observational cross-sectional and was carried out at the Tema Port Clinic, Ghana from February 2013 to August 2013. A total of 161 individuals comprising 100 persons with type 2 diabetes and 61 BMI-matched apparently healthy staff/workers (controls) from the Clinic, were recruited into the study. Sample size of 60 persons was adequate for this study assuming an odds ratio of 2.0 among diabetic subjects for dyslipidemia, at a 5% significant level and a power of 80%. An oral glucose tolerance test (FBG ≤ 6.9 mmol/L and a 2-h post-prandial ≤ 11.1 mmol/L), regarded as diagnostic [2] was performed on all volunteers. The ethical and protocol review committee of Narh-Bita College, Tema, reviewed and approved the study. Detailed explanations on purpose of the study, risk and benefits were made known to participants. In addition to the patient’s clinical data retrieved from medical records, a standard questionnaire was used to collect socio-demographic data from consented participants. Height was measured using a wall-mounted stadiometer (Secca, Germany). Body weight was by a standard digital scale (Tanita Corporation, Tokyo, Japan). Body mass index was calculated as weight divided by squared height (kg/m^2^). Blood pressure was taken using a mercury sphygmomanometer and stethoscope after participants had rested for 15 min. Participants was seated on a chair and the feet firmly rested on the floor. The measurement was taken from the same hand (left) rested on a desk with the antecubital fossa level with the palm facing upwards. High blood pressure was defined as SBP > 140 mmHg or DBP > 90 mmHg or self-reported controlled treatment using hypertensive medication [20]. Persons with gestational diabetes, human immunodeficiency virus (HIV), Hepatitis B infection, habitual smokers and subjects with stroke or amputation were excluded from the study.

Venous blood (4 mL) was obtained from the subjects between 07:00 and 09:00 h each day, after an overnight fast, according to Helsinki protocol declaration [21]. One milliliter of whole blood was transferred into sodium fluoride containing tube and centrifuged, with resulting plasma separated for the estimation of FBG. The remaining blood sample was placed into serum separator tube and processed. Serum was stored at − 20 °C until required for analysis. Samples were thawed for total cholesterol (T. Chol), triglycerides (TG) and high-density lipoprotein (HDL) estimations. All analysis were performed using auto-analyzer (Roche-Hitachi Modular Analytics, Tokyo, Japan). Cardiovascular risk was computed as the ratio of T. Chol to HDL and has been described by Wilson et al. [22]. Low-density lipoprotein cholesterol (LDL) was computed as previously described [23]. Liver markers including total proteins (TP), albumin (ALB), aspartate transaminase (AST), alanine transaminase (ALT), gamma-glutamyl transferase (GGT), alkaline phosphatase (ALP), total bilirubin (TBil) and direct bilirubin (DBil) were also analyzed (Roche-Hitachi Modular Analytics, Tokyo, Japan). Dyslipidemia was defined as: T. Chol (> 5.0 mmol/L), LDL-C (> 3.0 mmol/L), TG (> 1.7 mmol/L) and HDL (< 1.0 mmol/L for men and < 1.2 mmol/L for women) [24].

Data were expressed as mean ± standard deviation and in percentages where appropriate. The Statistical Package for Social Sciences (SPSS) version 20.0 (SPSS Software, San Diego, U.S.A.) was used for analysis. Unpaired student’s t-test was used to evaluate differences between two means. Pearson’s correlation coefficient (r) was used to establish relationship between two continuous variables. Statistical significance was set at p < 0.05 for all tests.

### Results

The clinical and biochemical parameters of the study population are shown in Table 1. The mean ages for persons with diabetes and controls were 51.67 and 42.05 years respectively and were statistically different (*p *< 0.0001). Systolic blood pressure (SBP), diastolic blood pressure (DBP), FBG, T. Chol, TG and ALT levels were respectively higher in persons with type 2 diabetes compared with controls (*p* > 0.05). There were however, no difference (*p* > 0.05) respectively, for BMI, GGT, HDL, ALP, TP and ALB levels between the two study groups. The inter-dependency of several parameters (Age, SBP, DBP, FBG, TG, LDL, T. Chol, TB, ALT and AST) within persons with type 2 diabetes is shown in Table 2. Age correlated negatively with SBP (r = − 0.260, p < 0.01) whiles positive relationships were found for TG vs FBG (r = 0.289, p < 0.01); ALT vs age (r = 0.388, p < 0.01) and AST vs TG (r = 0.248, p < 0.01).

**Table 1:** Clinical and biochemical variables of the study population

Variables	Type 2 diabetes (N = 100)	Controls (N = 61)	95% CI	*p*-value (*t*-test)
Age (years)	51.67 ± 9.25	42.05 ± 7.96	− 12.85 to (− 6.39)	< 0.0001
BMI (kg/m^2^)	28.37 ± 5.72	28.23 ± 4.84	− 7.35 to 4.97	0.9241
SBP (mmHg)	143.48 ± 24.97	135.16 ± 19.98	− 15.71 to (− 0.92)	0.0288
DBP (mmHg)	83.37 ± 15.06	74. 54 ± 13.51	47.17 to 56.41	0.0001
FBG (mmol/L)	11.62 ± 7.81	5.03 ± 0.61	− 8.56 to (− 4.62)	< 0.0001
T. Chol (mmol/L)	6.13 ± 0.70	4.58 ± 0.65	− 1.76 to (− 1.33)	< 0.0001
TG (mmol/L)	1.57 ± 1.01	1.04 ± 0.43	− 0.79 to (− 0.26)	0.0001
HDL (mmol/L)	1.26 ± 0.48	1.26 ± 0.47	− 0.15 to 0.15	1.0000
LDL (mmol/L)	3.68 ± 0.96	2.85 ± 0.86	− 1.12 to (− 0.53)	0.0001
Cardiovascular risk	4.88 ± 1.46	3.54 ± 1.38	− 1.79 to (− 0.88)	0.0001
Atherogenic index	5.89 ± 1.02	3.18 ± 0.08	− 2.96 to (− 2.45)	< 0.0001
Total Bil (μmol/L)	11.82 ± 8.43	13.75 ± 4.97	− 0.40 to 4.26	0.1065
Direct Bil (μmol/L)	4.42 ± 2.13	4.35 ± 2.53	− 0.79 to 0.65	0.8509
Indirect Bil (μmol/L)	7.72 ± 4.00	9.39 ± 3.92	0.40 to 2.93	0.0105
GGT (U/L)	30.45 ± 18.28	30.43 ± 11.58	− 5.04 to 5.20	0.9756
ALT (U/L)	35.04 ± 21.28	27.85 ± 9.49	− 12.85 to (− 1.53)	0.0138
AST (U/L)	32.60 ± 6.31	30.36 ± 7.27	− 3.93 to (− 0.54)	0.0805
ALP (U/L)	182.82 ± 64.57	177.50 ± 52.69	− 24.54 to 13.90	0.5882
Total protein (g/L)	65.77 ± 15.46	68.35 ± 10.06	− 1.774 to 6.934	0.2473
Albumin (g/L)	38.32 ± 7.24	38.60 ± 5.87	− 1.87 to 2.43	0.7990
Duration of DM (years)	3.53 ± 4.72	–	–	–

Values are given as mean ± SD. Atherogenic index = [(TC − HDL)/HDL], Cardiovascular risk = [TC/HDL], p < 0.05 is statistically significant

*SBP* systolic blood pressure, *DBP* diastolic blood pressure, *BMI* body mass index, *T. Chol* total cholesterol, *TG* is triglyceride, *HDL* high-density lipoprotein, *LDL* low-density lipoprotein, *FBG* fasting blood glucose, *AST* aspartate transaminase, *ALT* alanine transaminase, *GGT* gamma-glutamyl transferase, *ALP* alkaline phosphatase, *Bil* bilirubin, *DM* diabetes mellitus

**Table 2:** Correlation matrix of selected parameters in persons with 2 diabetes

	Age	SBP	DBP	FBS	TG	LDL	T. Chol	TB	IB	ALT	AST
Age	1.000										
SBP	−*0.260*	1.000									
DBP	−0.091	*0.642*	1.000								
FBS	0.112	0.191	− 0.013	1.000							
TG	0.014	0.120	− 0.009	*0.289*	1.000						
LDL	−0.034	0.020	− 0.041	0.124	− 0.070	1.000					
T. Chol	0.078	0.107	0.110	− 0.032	0.011	*0.297*	1.000				
TB	0.009	− 0.031	0.045	0.005	0.081	− 0.005	− 0.055	1.000			
IB	0.011	0.059	0.079	0.005	0.094	0.005	− 0.051	*0.921*	1.000		
ALT	*0.388*	− 0.058	− 0.124	0.091	0.025	− 0.006	− 0.005	− .007	0.040	1.000	
AST	0.133	− 0.024	− 0.067	− 0.078	*0.248*	0.099	− 0.017	0.100	0.118	− 0.008	1.000

Values in italic is significant (p < 0.05)

*SBP* systolic blood pressure, *DBP* diastolic blood pressure, *T. Chol* total cholesterol, *TG* triglyceride, *LDL* low-density lipoprotein, *FBG* fasting blood glucose, *AST* aspartate transaminase, *ALT* alanine transaminase, *TB* total bilirubin, *IB* indirect bilirubin

### Discussion

This study sought to investigate dyslipidemia in Ghanaian persons with type 2 diabetes. In this study, T. Chol, TG and their derived fractions, were significantly higher in persons with type 2 diabetes compared with controls. This lipid pattern was consistent with other studies implicating dyslipidemia in type 2 diabetes [25, 26]. Although the mechanisms are not fully elucidated, defects in insulin action and hyperglycemia have been shown to alter plasma levels of lipoprotein in patients with type 2 diabetes [27]. The prevalence of hypercholesterolemia in this study was 53% (not shown). Others have shown prevalence ranging from 30 to 80% [8, 28–31]. The presence of elevated levels of cholesterol is known to play a key role in both the initiation and progression of atherosclerosis, as well as in other clinical consequences such as myocardial infarction, stroke, peripheral vascular disease, and heart failure [32, 33].

Fasting blood glucose levels associated positively and significantly with triglyceride. In type 2 diabetes, there is both insulin insensitivity and resistance resulting in impaired glucose availability in cells to provide energy. This may be responsible for increased lipolysis resulting in influx of free fatty acids to the liver, which may drive up hepatic triglyceride synthetic rates evidenced in type 2 diabetes [34]. Elevated TG and reduced levels of HDL has been implicated in liver dysfunction and may be attributed to accumulated fatty liver [35, 36]. Improved glycemic control is generally associated with favourable lipoprotein levels and reductions in both TG and T. Chol levels [27, 28] via mechanisms that decrease circulating very low density lipoproteins [37] and the up regulation of LDL catabolism [38, 39].

The development of lipid abnormalities and cardiovascular diseases in diabetes mellitus have also been suggested to be predicted by several other factors including raised BMI, hypertriglyceridemia, low HDL levels and hypertension. This study however, did not observe significant relationships with several of the above-mentioned metabolic parameters. Non-differences observed could be attributable to different volunteer characteristics and the sampling technique used in subject recruitment which may have introduced a bias. Differences observed for both systolic and diastolic blood pressures between the two study groups was however significant. Chronic glycemic status tend to be associated with increases in both systolic and diastolic blood pressures [40, 41].

In summary, results highlight dyslipidemia in persons with type 2 diabetes with increased circulating lipids affecting liver health. Proper management of diabetes mellitus including lifestyle modifications can reduce dyslipidemia and the risk of cardiovascular disease and liver dysfunction.

## Limitations

This study was not without limitations. This study did not measure plasma insulin concentrations which would have enabled the authors explore associations with insulin resistance. Our data only provide observation of associations but not causality and cannot be generalized to all populations. In addition to larger sample size, future work should consider the role of dyslipidemia in diabetic complications.

## Abbreviations

FBG: fating blood glucose
TG: triglyceride
ALT: alanine transaminase
AST: aspartate transaminase
HDL: high density lipoprotein
VLDL: very low-density lipoprotein
LDL: low density lipoprotein
CR: coronary risk
CVD: cardiovascular disease
T. Chol: total cholesterol
BMI: body mass index
SBP: systolic blood pressure
DBP: diastolic blood pressure
ALB: albumin
TP: total protein
GGT: gamma-glutamyl transferase
ALP: alkaline phosphatase
HIV: human immunodeficiency virus
